# Unravelling HetC as a peptidase-based ABC exporter driving functional cell differentiation in the cyanobacterium *Nostoc* PCC 7120

**DOI:** 10.1128/spectrum.04058-23

**Published:** 2024-02-15

**Authors:** Raphaël Rachedi, Véronique Risoul, Maryline Foglino, Yanis Aoudache, Kevin Lang, Stéphanie Champ, Elise Kaplan, Cédric Orelle, Badreddine Douzi, Jean-Michel Jault, Amel Latifi

**Affiliations:** 1 Aix-Marseille Université, CNRS, Laboratoire de Chimie Bactérienne LCB, IMM, Marseille, France; 2 Université de Lorraine, INRAE, DynAMic, Nancy, France; 3 Microbiologie Moléculaire et Biochimie Structurale, UMR5086 Université de Lyon/CNRS, IBCP, Lyon, France; South China Sea Institute of Oceanology Chinese Academy of Sciences, Guangzhou, China

**Keywords:** ABC-transporter, cyanobacteria, gene regulation, heterocyst differentiation, peptidase, PCAT, signaling

## Abstract

The export of peptides or proteins is essential for a variety of important functions in bacteria. Among the diverse protein-translocation systems, peptidase-containing ABC transporters (PCAT) are involved in the maturation and export of quorum-sensing or antimicrobial peptides in Gram-positive bacteria and of toxins in Gram-negative organisms. In the multicellular and diazotrophic cyanobacterium *Nostoc* PCC 7120, the protein HetC is essential for the differentiation of functional heterocysts, which are micro-oxic and non-dividing cells specialized in atmospheric nitrogen fixation. HetC shows similarities to PCAT systems, but whether it actually acts as a peptidase-based exporter remains to be established. In this study, we show that the N-terminal part of HetC, encompassing the peptidase domain, displays a cysteine-type protease activity. The conserved catalytic residues conserved in this family of proteases are essential for the proteolytic activity of HetC and the differentiation of heterocysts. Furthermore, we show that the catalytic residue of the ATPase domain of HetC is also essential for cell differentiation. Interestingly, HetC has a cyclic nucleotide-binding domain at its N-terminus which can bind ppGpp *in vitro* and which is required for its function *in vivo*. Our results indicate that HetC is a peculiar PCAT that might be regulated by ppGpp to potentially facilitate the export of a signaling peptide essential for cell differentiation, thereby broadening the scope of PCAT role in Gram-negative bacteria.

**IMPORTANCE:**

Bacteria have a great capacity to adapt to various environmental and physiological conditions; it is widely accepted that their ability to produce extracellular molecules contributes greatly to their fitness. Exported molecules are used for a variety of purposes ranging from communication to adjust cellular physiology, to the production of toxins that bacteria secrete to fight for their ecological niche. They use export machineries for this purpose, the most common of which energize transport by hydrolysis of adenosine triphosphate. Here, we demonstrate that such a mechanism is involved in cell differentiation in the filamentous cyanobacterium *Nostoc* PCC 7120. The HetC protein belongs to the ATP-binding cassette transporter superfamily and presumably ensures the maturation of a yet unknown substrate during export. These results open interesting perspectives on cellular signaling pathways involving the export of regulatory peptides, which will broaden our knowledge of how these bacteria use two cell types to conciliate photosynthesis and nitrogen fixation.

## INTRODUCTION

Transporters belonging to the ATP-binding cassette (ABC) superfamily couple the import or export of a wide spectrum of substrates to the hydrolysis of adenosine triphosphate (ATP), a process that energizes the translocation process (1). They are characterized by a high conservation of the nucleotide-binding domains (NBDs) which contrast with the high variability of the transmembrane domains (TMDs) that form the translocation pathway (1, 2). The most recent classification of ABC transporters is based on the structural determinants of these TMDs (3).

Some members of this superfamily export peptides or proteins concomitantly with their cleavage; they have been designated as “Peptidase Containing ABC Transporters” (PCATs) (4). Their N-terminal domain encodes a C39 class of cysteine proteases (Interpro:IPR005897) that excises a leader sequence typically ending with a double Glycine motif (Gly-Gly, Gly-Ser, or Gly-Ala) (5–7). PCATs are involved in the export of bacteriocins (5, 6) and pheromones (7) in Gram-positive bacteria. In Gram-negative bacteria, they have been found to associate with a membrane fusion protein and an outer membrane factor to export processed toxins (4, 8). The structure of a full-length PCAT from the Gram-positive bacterium *Clostridium thermocellum* revealed that, in the absence of ATP, the NBD and the peptidase domains interact. ATP binding and hydrolysis release the peptidase domain, therefore coupling the proteolytic cleavage of the substrate to its export (9).

Based on the conservation of gene orthologs, it is probable that PCATs in Gram-negative bacteria have functions beyond toxin export despite the absence of evidence to support this assertion. For instance, the protein HetC, which can be classified in the PCAT group based on sequence similarities, is essential for heterocyst differentiation in the filamentous and diazotrophic cyanobacterium *Anabaena*/*Nostoc* (hereafter *Nostoc*) PCC 7120 (4, 10). *Nostoc* grows in long filaments of photosynthetic cells when combined nitrogen (nitrate or ammonium) is abundant but if this element becomes limiting, 5%–10% of the vegetative cells differentiate into heterocysts which are microoxic and non-dividing cells that host the oxygen-sensitive nitrogenase (11–13).

The transcription of the *hetC* gene is induced early during the differentiation process (10, 14). A mutant strain lacking *hetC* is unable to grow under diazotrophic conditions, does not form mature heterocysts, and exhibits patterned weakly fluorescent cells (10). Contrary to heterocysts, proheterocysts of the *hetC* mutant maintain cell division, which is why HetC has been proposed to regulate the transition to a non-dividing terminal state during heterocyst development (15). The transcription level of the cell division gene *ftsZ* and of several genes involved in the differentiation process is modified in the proheterocysts of the *hetC* deletion mutant (16), but the functional link between HetC and the transcriptional regulation of these genes is still unknown. Heterocyst formation was reported to be abolished in a *hetC* mutant lacking the peptidase domain (17), suggesting that it is important for HetC function. Nevertheless, it is yet to be proven if HetC functions as a PCAT transporter and if this function underlies HetC role in cell differentiation.

In this study, we established the topology of HetC and showed that its peptidase domain acts as a cysteine protease. The catalytic amino acids of the peptidase and ATPase domains were found to be essential for cell differentiation, suggesting that it is the transporter function of HetC that may be essential to this process. In addition, our results demonstrate that the cyclic nucleotide-binding domain (cNMP) identified in the N-terminal region of HetC is crucial for heterocyst formation and is involved in ppGpp binding. Finally, based on the knowledge of PCATs, we discuss a functional model explaining the role of HetC in the developmental process.

## RESULTS

### Determination of the membrane topology of HetC

**Fig 1:**
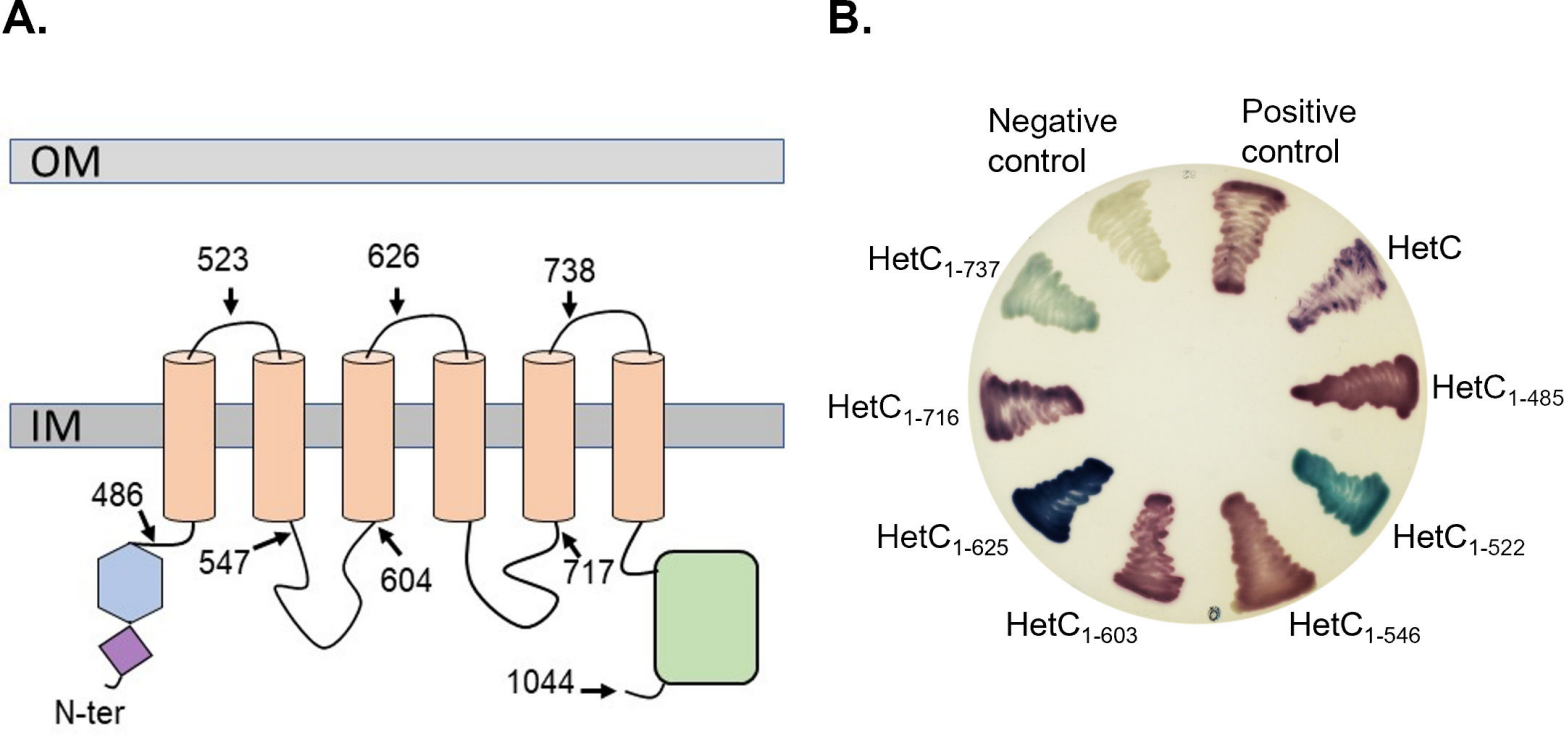
Membrane topology analysis of HetC protein. (**A**) Full-length HetC or truncated versions were fused in frame to the dual reporter phoA-lacZ in the pKTop vector. The diagram schematizes HetC sequence with the predicted cyclic nucleotide-binding domain (purple), C39-peptidase domain (blue), transmembrane helices (salmon), and ATP-binding cassette domain (green). The C-terminal residue of each HetC variant is indicated. (**B**) *Escherichia coli* DH5α transformants producing the different HetC-PhoA-LacZ fusions were plated on LB agar plates supplemented with the chromogenic substrates: X-Pho for PhoA and Red-GAL for LacZ. Red colonies indicate that the PhoA-LacZ fusion localizes in the cytoplasm, whereas the blue color indicates that the PhoA-LacZ fusion is present in the periplasm space. The controls consist of *Escherichia coli* DH5α transformed with either a pET28 (negative control) or the pKTop vector (positive control for cytoplasmic localization).

HetC is predicted to be an inner membrane protein possessing two soluble N- and C-terminal domains. Defining HetC topology is a prerequisite to understand its structure-function relationship, we, therefore, decided to determine it experimentally using the dual *pho-lac* reporter tool (18). In this system, a periplasmic location of the reporter is revealed by a blue color induced by the alkaline phosphatase activity. If the reporter is located in the cytoplasm, the colonies appear red due to alpha complementation of the β-galactosidase activity. In order to select the positions in HetC sequence where the dual reporter should be fused, we combined the *in silico* topology predictions obtained from CCTOP, which are based on the comparison of multiple different methods (19), with the 3D-model of HetC predicted by Alphafold (20) (Fig. S1B; Fig. 1A). This allowed us to identify the predicted TMHs (transmembrane helices) on HetC structure and to guide the selection of the insertion positions for the double reporter to avoid destabilization of the protein secondary structures and/or topology. The full-length HetC (HetC_1–1044_) and seven HetC fragments truncated at the positions indicated in Fig. 1A (HetC_1–485_, HetC_1–523_, HetC_1–547_, HetC_1–604_, HetC_1–626_, HetC_1–717_, HetC_1–738_) were fused at their C-terminal ends to the dual reporter. As shown in Fig. 1B, HetC full-length and HetC_1–486_ displayed red phenotype indicating that the two soluble N- and C-terminal domains are cytoplasmic. Analysis of the six other constructs reveals that HetC possesses six transmembrane helices with two loops connecting the transmembrane domains located in the cytoplasm and three in the periplasm. The experimentally established topology of HetC is, thus, consistent with those of PCAT exporters.

### HetC N-terminal domain displays a cysteine protease activity

**Fig 2:**
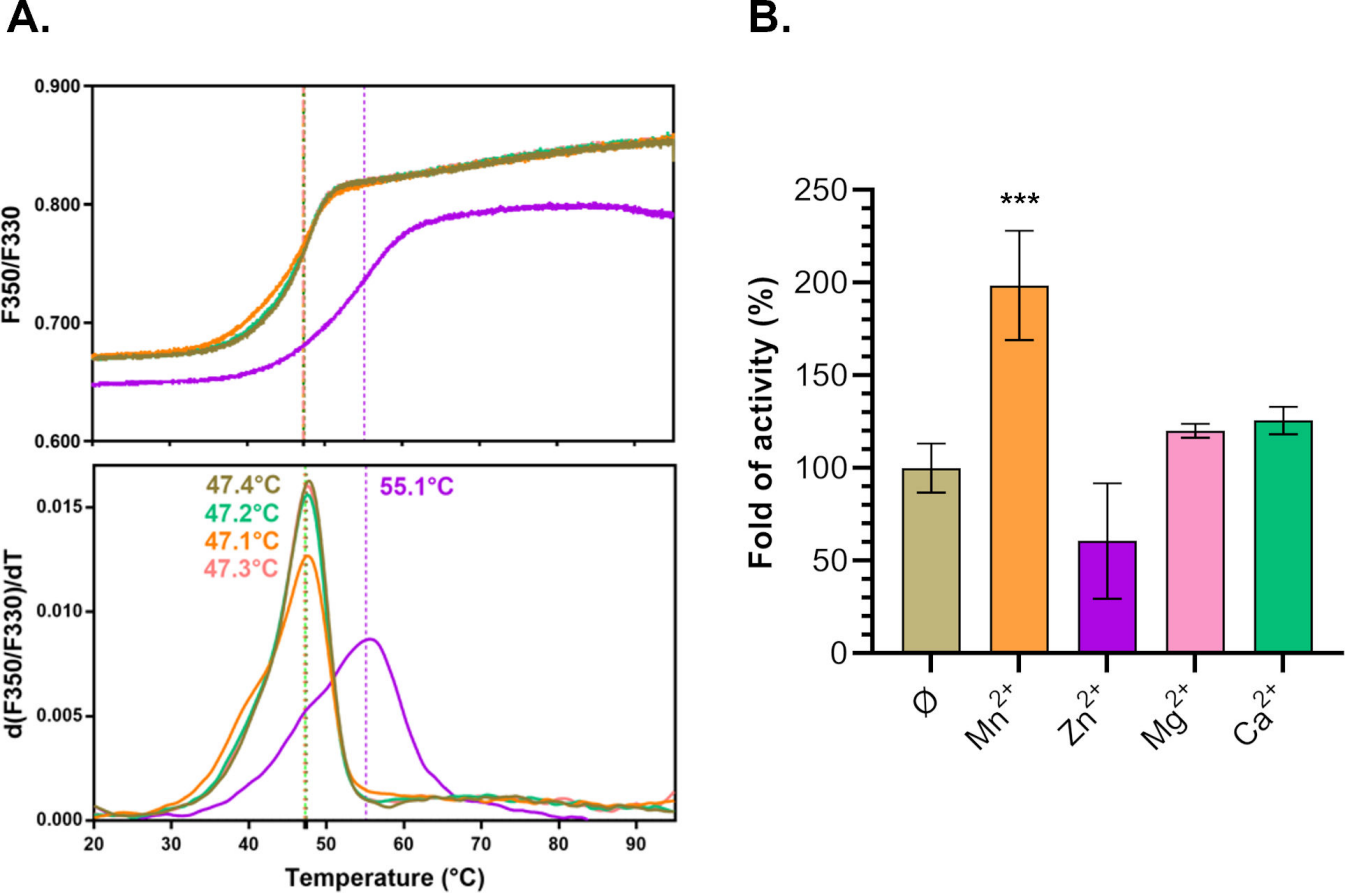
Characterization of HetC putative C39 peptidase domain. (**A**) Thermal stability of HetCNTD wild-type and effect of ZnCl2. The ratio of fluorescence emitted at 350 and 330 nm and first-derivatives with *T*_m_ are shown for the HetCNTD wild-type (upper and lower panels). The proteins were incubated at 1 mg/mL in the absence (olive curve) or in the presence of 1 mM ZnCl_2_ (violet), MnCl_2_ (orange), MgCl_2_ (pink), or CaCl_2_ (green). The curves represent the mean of two replicates for each condition (∆*T*_m_ ≤ 0.45°C, except for 5 mM ZnCl_2_ with a ∆*T*_m_ ≤ 2.2°C). (**B**) Comparison of the protease activity of the purified HetCNTD in the presence of different cations (5 mM). Results are expressed in fold of activity (%) relative to the mean of wild-type HetCNTD from three independent repetitions. *** indicates the *t*-test value (see Materials and Methods).

If HetC is, indeed, a member of the PCAT transporters family, it should possess a proteolytic activity typical of this class of proteins. To test this hypothesis, we purified the soluble N-terminal domain (HetC_NTD_) (Fig. S2) and analyzed its thermal stability in the presence of several cations (Mg^2+^, Mn^2+^, Zn^2+^, and Ca^2+^). As shown in Fig. 2A, the protein melting temperature increased by 8° in the presence of Zn^2+^ from ∼47 to 55°C. This cation likely associates with the NTD of HetC leading to its stabilization. We then analyzed the ability of this domain to cleave L-Arginine *p*-nitroanilide, which is an artificial substrate of the cysteine protease family. This approach was successfully used for the CvaB transporter, which belongs to the same family as HetC, and has allowed the identification of the catalytic residues important for the proteolytic and transport activities (8). In the presence of the N-terminal domain of HetC, the L-Arginine *p*-nitroanilide was cleaved into *p*-nitroanilide, and the specific activity of the protein was low 0.22 ± 0.08 pmol/min/µg of protein. The addition of Zn^2+^ had no effect on the proteolytic activity (Fig. 2B), suggesting that it is not involved in the catalytic reaction. A twofold increase of the activity was observed in the presence of Mn^2+^ (Fig. 2B). As CvaB is a calcium-dependent protease, we measured the activity of HetC peptidase domain in the presence of this cation, but no effect was observed. To further confirm that HetC belongs to the PCAT family, we substituted the conserved cysteine and histidine catalytic residues of the peptidase domain [C347 and H420 in HetC (8)] into alanine.

**Fig 3:**
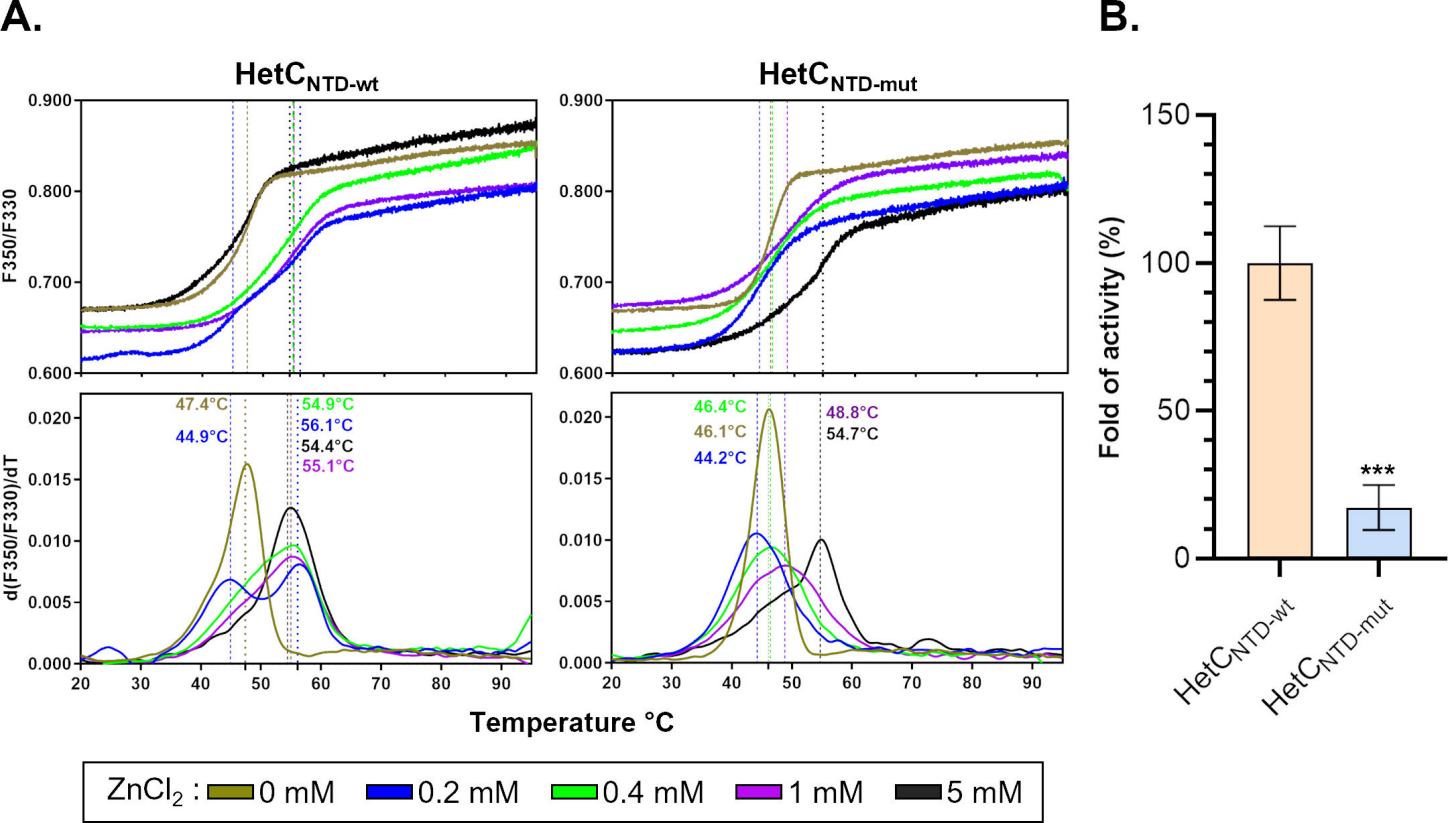
Characterization of HetC putative C39 catalytic residues. (**A**) Thermal stability of HetCNTD wild-type or mutant domains and effect of ZnCl2. The conditions were similar to those used in Fig. 2. Ratio of fluorescence emitted at 350 and 330 nm and first derivatives with *T*_m_ are shown (upper and lower panels, respectively) for the HetCNTD wild-type protein (left panels) or mutant protein (right panels). The proteins were incubated at 1 mg/mL in the absence (olive curve) or in the presence of 0.2 mM ZnCl_2_ (blue), 0.4 mM ZnCl_2_ (green), 1 mM ZnCl_2_ (violet), and 5 mM ZnCl_2_ (black). The curves represent the mean of two replicates for each condition (∆*T*_m_ ≤ 0.45°C, except for 5 mM ZnCl_2_ with a ∆*T*_m_ ≤ 2.2°C). (**B**) Comparison of the specific activity of HetC (HetCNTD-wt) and HetCC347A/H420A (HetCNTD-mut) N-terminal domains. Results are expressed in fold of activity (%) relative to the mean of HetCNTD-wt from three independent repetitions. *** indicates the *t*-test value (see Materials and Methods).

To verify that the resulting variant (HetC_NTD-mut_) conserved its structural integrity, we compared its thermal stability to that of the wild-type construct (HetC_NTD-wt_). Both proteins showed similar melting temperatures (Fig. 3A). Yet, the mutant protein seems to bind Zn^2+^ with a reduced affinity as compared to the wild-type protein because lower concentrations of this cation (e.g., 0.4 mM) only protect the wild-type protein against early thermal denaturation. A similar protection was nevertheless observed in the presence of 5 mM Zn^2+^ for both proteins. As the addition of zinc did not affect peptidase activity, zinc cation may have a structural rather than a catalytic role. Proteolytic assays showed that the substitutions of the catalytic residues significantly impaired the proteolytic activity, as the activity was reduced by 80% as compared to HetC_NTD-wt_ (Fig. 3B). Altogether, these results indicate that HetC is a cysteine-peptidase whose *in vitro* proteolytic activity is enhanced in the presence of Mn^2+^.

### The peptidase and ATPase activities of HetC are required for heterocyst differentiation

**Fig 4:**
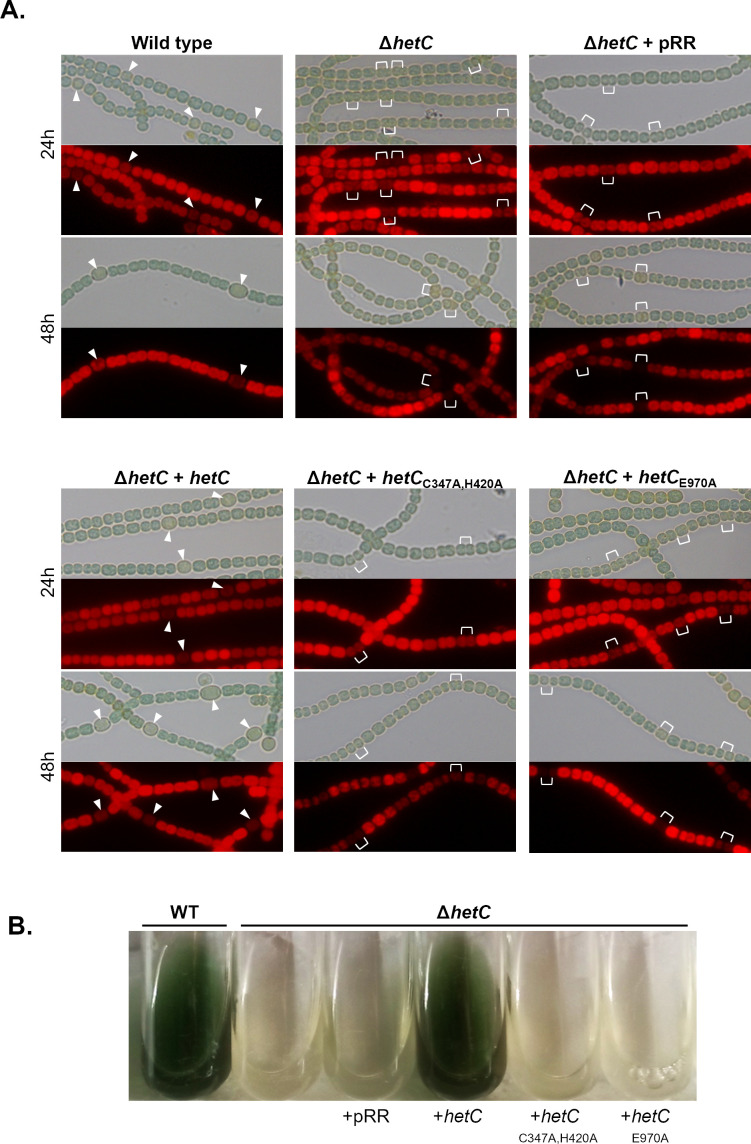
Phenotypic and growth assessment of the role of HetC putative C39 peptidase and ATPase domains catalytic residues in heterocyst formation. (**A**) The hetC deletion strain was conjugated with unmodified pRR001 vector (pRR) or with derivatives encoding wild-type hetC and hetC with mutations impairing the C39 peptidase catalytic residues (C347→A /H420→A) or the ATPase domain catalytic residue (E970→A) under the control of the copper inducible PpetE promoter. Observations were made at 24 and 48 h after nitrogen stepdown. Top, bright field; bottom, TRITC fluorescence. Representative heterocysts are indicated with white arrowheads, representative clusters of dividing pro-heterocysts are indicated with white brackets. (**B**) ΔhetC strain and ΔhetC strains bearing the unmodified pRR001 vector (pRR) (ΔhetC + pRR) or derivatives bearing hetC, hetCC347/H420A, or hetCE970A genes expressed from the PpetE copper inducible promoter were compared with the wild-type strain for survival in BG110.

To explore whether the presumable transport activity of HetC is required for cell differentiation, a marker-less mutant of *hetC* was constructed using a Cpf1-CRISPR approach as described previously (21). The *Δ*hetC mutant strain was unable to form mature heterocysts (Fig. 4A; Table 1), and a cluster of 2–3 cells with a weak fluorescence background was observed in the filaments of the mutant (Fig. 4A). Furthermore, the mutant did not grow under nitrogen-fixing conditions (Fig. 4B). It is concluded that, in the genetic background of our *Nostoc* strain, *hetC* is crucial for both heterocyst formation and growth under nitrogen-fixing conditions.

**TABLE 1:** Heterocyst percentages of the strains analyzed in this study^*a*^

	Total cells counted	Heterocysts %	SD %
WT	345	8.39	0.11
Δ*hetC*	1,110	0	–
Δ*hetC* + pRR	1,275	0	–
Δ*hetC + hetC*	1,889	8.21	0.66
Δ*hetC + hetC*_C347A/H420A_	1,461	0	–
Δ*hetC + hetC*_E970A_	1,856	0	–
Δ*hetC + hetC*ΔcNMP	1,601	0	–

^*a*^ Bacterial cultures were grown in BG11_0_ for 48 h. Heterocysts were counted in three independent images, and their average number was counted relative to the total number of cells counted. SD represents the standard deviation among heterocyst counts obtained from these images.

The effect of the gene deletion was complemented by the expression *in trans* of wild-type *hetC* (Fig. 4; Table 1) but not by *hetC* mutant genes encoding proteins substituted either in the peptidase catalytic residues (*hetC*_C347A/H420A_) or in the ATPase catalytic glutamate residue [hetC_E970A_ (22)]. In the later cases, only proheterocysts similar to those formed by the mutant strain were observed (Fig. 4A). Furthermore, the strains expressing these mutated versions of *hetC* were unable to grow in diazotrophic conditions (Fig. 4B), similarly to the *Δ*hetC mutant. These results indicate that both the peptidase and ATPase activities of HetC are essential for heterocyst development.

### The cyclic nucleotide-binding domain of HetC is required for cell differentiation

**Fig 5:**
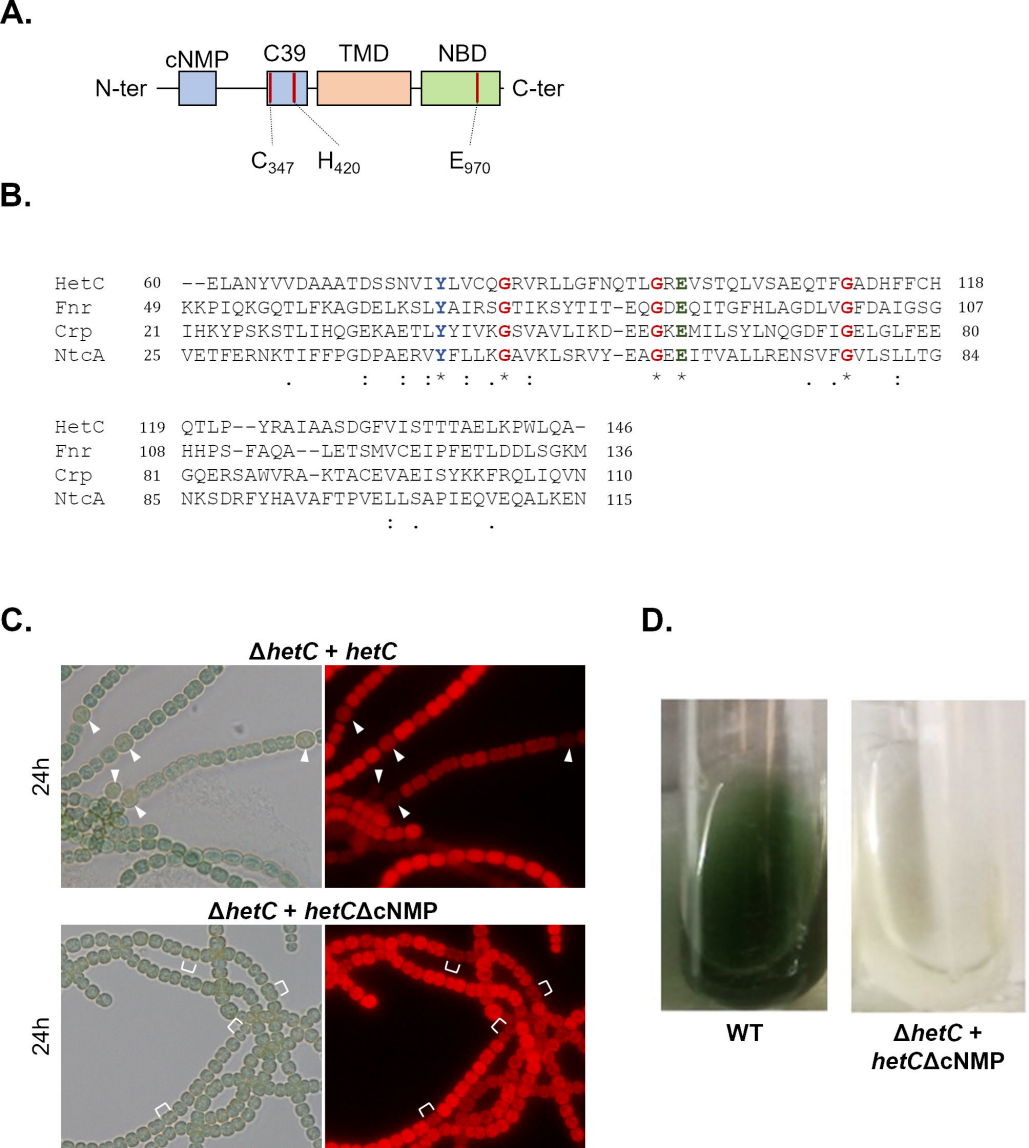
Characterization of HetC putative cNMP domain (IPR000595. PF00027). (**A**) Schematized HetC sequence with the predicted cNMP domain. (**B**) Alignment of the putative cNMP domain of HetC with similar domains of Fnr (*E. coli*), Crp (*E. coli*), NtcA (*Nostoc* PCC7120). The conserved glycine residues known to be essential to the structural integrity of the domain are marked in red. The position of the cNMP domain in each protein sequence, as given by Pfam, is indicated as follows: HetC, 60–146; Fnr, 49–136 Crp, 21–110; NtcA, 25–115. (**C**) Phenotypic assessment of the putative HetC cNMP domain role in heterocyst formation. The hetC deletion strain was conjugated with a pRR001 derivative encoding hetC ORF lacking the cNMP domain under the control of the copper inducible PpetE promoter. Observations were made under the microscope at 24 and 48 h after nitrogen stepdown without extra adjunction of cooper ion. Left panel, bright field; right panel, TRITC fluorescence. Representative heterocysts are indicated with white arrowheads; representative dividing pseudo-pro-heterocysts are indicated with white brackets. (**D**) Growth assessment of the wild-type strain and the hetC bearing hetCΔcNMP gene expressed from the PpetE copper inducible promoter after 7 days of combined nitrogen deprivation.

The N-terminal part of HetC contains a putative ligand-binding domain that is found in many transcriptional regulators (Fig. 5A and B). This domain is known as the cyclic nucleotide-binding domain (cNMP-binding) and is present in several distantly related proteins (23). The archetypes of proteins carrying this domain are the regulator of catabolic repression in multiple Gram-negative bacteria, CRP, which binds cAMP (24), and the transcriptional regulator NtcA which binds 2-oxo-glutarate (2-OG) to regulate the carbon-nitrogen balance in cyanobacteria (25, 26). In filamentous cyanobacteria, such as *Nostoc*, NtcA is a key regulator of heterocyst differentiation (27). Other potential ligands for this family of proteins include c-GMP and oxygen (23).

To investigate the importance of the cNMP domain in the protein function, a *hetC* truncated gene deleted from the region encoding this domain was constructed and expressed *in trans* in the *Δ*hetC strain. The resulting strain (*Δ*hetC/hetC*Δ*cNMP) was, as the *Δ*hetC strain, unable to form mature heterocyst (Fig. 5C) and to grow under nitrogen-fixing conditions (Fig. 5D) indicating that this domain is essential for the function of HetC.

**Fig 6:**
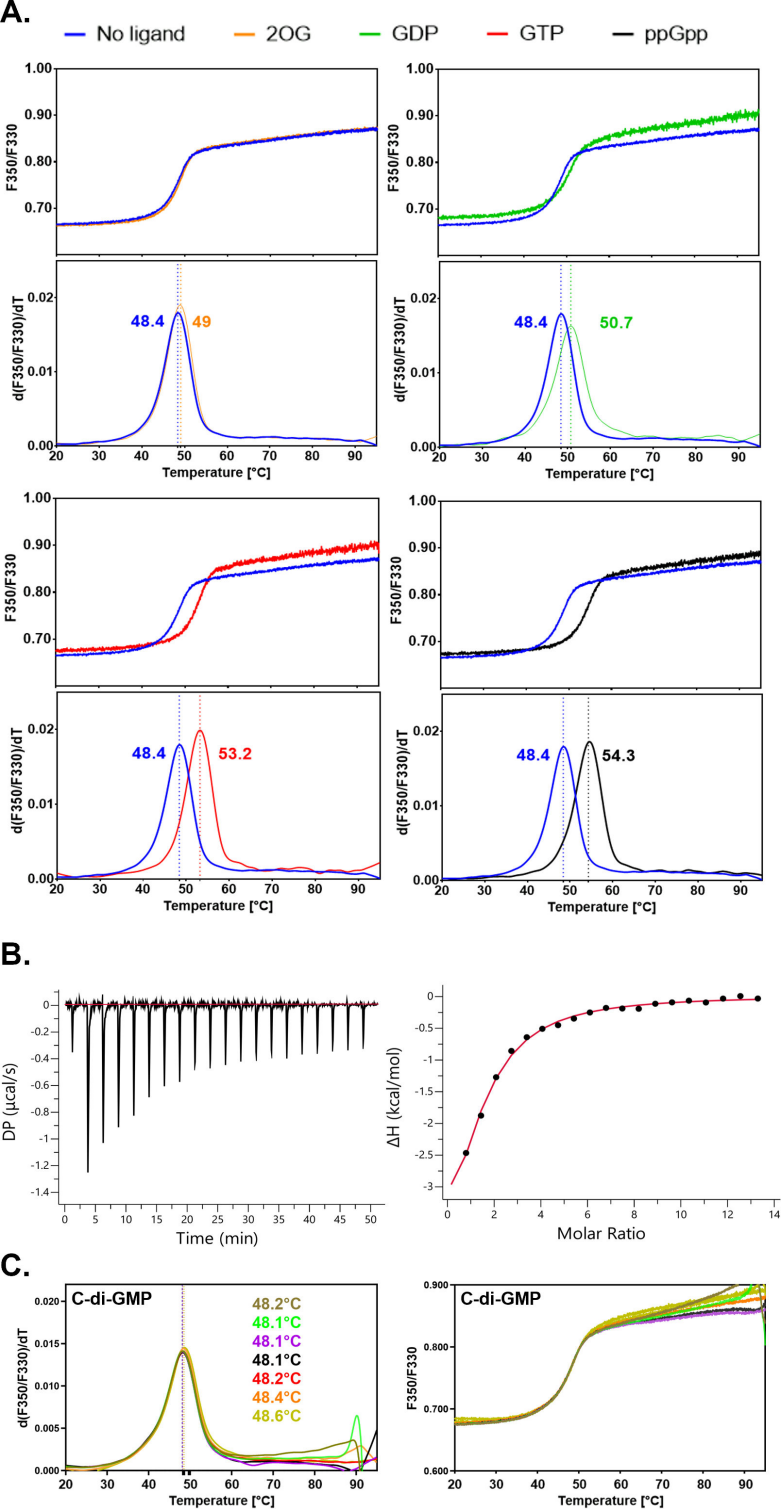
Binding of effectors to the HetCNTD. (**A**) Thermostability analysis of wild-type HetCNTD probed by differential scanning fluorimetry. The ratio of fluorescence emitted at 350 and 330 nm (top panels) and first-derivatives curves (bottom panels) indicating the *T*_m_ in °C are shown. Curves are colored as follows: HetCNTD in the apo state (blue) or in the presence of 5 mM 2-OG (orange), 5 mM GDP (green), 5 mM GTP (red), or 5 mM ppGpp (black). The curves correspond to an average of two identical samples with a ∆*T*_m_ ≤ 0.4°C between duplicates. (**B**) Representative ITC experiment demonstrating the interaction between the HetC N-terminal domain and ppGpp. The top panel shows background-corrected heats of ppGpp injection and the bottom panel a fitted binding curve of the data. Three ITC experiments were performed leading to a fitted KD of 59 ± 11 µM for the triplicate and 52 µM for the curve shown.

To determine the potential role of this domain, we investigated whether one of the known ligands of this protein family could protect the NTD of HetC against thermal denaturation using nanoDSF. None of the canonical ligands described so far for the proteins containing a cNMP domain, i.e., cyclic nucleotide such as cAMP or 2-OG, were capable to induce a significant protection against thermal denaturation of HetC_NTD_ (Fig. 6A). We, therefore, expanded our analysis to other compounds known to bind ligand-binding domains (22). The addition of GDP or GTP to HetC_NTD_ provided significant protection (∆*T*_m_ = 2.3°C and ∆*T*_m_ = 4.8°C, respectively), while the addition of ppGpp provided an even greater protection (∆*T*_m_ = 5.9°C) (Fig. 6A). The binding of ppGpp to HetC_NTD_ was confirmed by ITC experiment with an estimated *K*_D_ ~59 ± 11 µM (Fig. 6B). The role of ppGpp in the control of bacterial physiology and metabolism is well established so far. It mediates the stringent response, which is a global regulatory process induced under nutrient and energy limitation, and environmental stresses (28). It is possible that ppGpp might regulate HetC activity *in vivo*. We further investigated whether HetC_-NTD_ could bind a variety of other potential ligands, one of which was c-di-GMP; however, our results showed no interaction (as shown in Fig. S3). To summarize, we tested HetC_NTD_ against an extensive range of ligands, including XTP, UTP, CTP, TTP, ATP, GMP-PNP, AMP-PNP, GDP, ADP, 2-OG, c-di-GMP, cCMP, and cAMP. Out of all these, ppGpp demonstrated the best ability to bind to HetC_NTD_ in the context of our experimental settings (Fig. S3).

## DISCUSSION

The *hetC* gene was identified by a genetic screen aimed at identifying mutants unable to differentiate heterocysts in *Nostoc* (10). The phenotype of *hetC* mutants was found to be influenced by genetic diversity among *Nostoc* strains cultivated in different laboratories (29). In a comparative study, one of the three studied *hetC* mutant strains showed the formation of proheterocyt clusters, while another strain displayed heterocysts, and the third showed both proheterocysts and heterocyst formation (29). In our study, we observed that the deletion of the *hetC* gene resulted in the loss of the capacity of the strain to form mature heterocysts (Fig. 3; Table 1). The mutant filaments exhibited cell clusters with diminished autofluorescence, reminiscent of proheterocysts. The size of these cells was not significantly reduced in comparison to the vegetative cells unlike the proheterocysts in the initially described *hetC* mutant (15). This discrepancy could be due to genetic differences between the two original strains or possibly because the proheterocysts in our *hetC* mutant were mostly not undergoing division at the time the images were captured. Regardless of the type of *hetC* mutants reported, whether they formed a cluster of proheterocysts (15), a reduced number of heterocysts (23, 30), or no (pro)heterocysts at all (17), all of them were unable to sustain growth under nitrogen-fixing conditions. This highlights the crucial role of HetC in functional heterocyst formation.

Since its discovery, HetC has been designated as an ABC exporter with a C39 peptidase domain, but this was based only on sequence similarities. This family of exporters is divided into two subgroups depending on whether the peptidase domain can be predicted as being functional or not. The first subgroup contains members that possess a typical peptidase domain with the conserved catalytic residues required for substrate cleavage. The second subgroup comprises members with a degenerate peptidase domain lacking the catalytic cysteine residue and, therefore, incapable of proteolytic activity. This latter subgroup is known as C39-like domain (31). Whether or not it has proteolytic activity, the peptidase domain is required for the interaction of the transporter with its substrate (32). A key element in understanding the transport mechanism of these proteins is, therefore, the characterization of their peptidase domain. A comparative sequence analysis of HetC showed the presence of the catalytic cysteine and histidine residues that are characteristic of PCATs (Fig. 5A). By using a substrate typical of C39 family proteases, we found that the N-terminal part of HetC which includes this peptidase domain has a proteolytic activity *in vitro* (Fig. 2B). This activity was strongly affected when the catalytic residues were substituted into alanine (Fig. 3B). Taken together, these results confirm that HetC belongs to the family of cysteine proteases. The specific activity obtained with the N-terminal domain of HetC was sevenfold higher than the activity of the apo-CvaB peptidase domain which is, to the best of our knowledge, the only other protein belonging to the PCAT family of Gram-negative bacteria that was analyzed using the same substrate (8). *In vivo*, the peptidase catalytic residues of HetC were found to be essential for heterocyst formation (Fig. 4), suggesting that the proteolytic activity is involved in the control of this cellular process. In addition, as the substitution of the catalytic residue of the transporter nucleotide-binding domain (NBD) also abolished cell differentiation, and because this activity is a well-established characteristic of ABC transporters (22), we concluded that HetC belongs to the PACT family. Furthermore, the topology determined for HetC is in line with this conclusion (Fig. 1). The peptidase and the NBD domains were located in the cytoplasm which is consistent with the interaction with a cytosolic substrate, its cleavage, and its subsequent translocation through the inner membrane. It has been proposed that HetC might translocate its substrate to the periplasm rather than to the extracellular environment (33). This hypothesis is supported by the fact that no gene encoding a membrane fusion protein is located in the vicinity of *hetC*, contrary to its homologs in other genomes (4).

Interestingly, in addition to the peptidase domain, the N-terminal part of HetC includes a typical cNMP domain, the deletion of which abolished HetC function (Fig. 5). This domain is known to bind various effectors (cAMP, cGMP, O_2_, 2-OG) (23). The ppGpp nucleotide was found to bind HetC_NTD_ with a *K*_d_ of 59 µM (Fig. 6). Given that the precise concentration of ppGpp in cyanobacteria is currently unknown, it is difficult to determine the physiological significance of the *K*_d_ value presented in this study. The cNMP domains are largely conserved among prokaryotic transcriptional regulators. HetC does not belong to this class of proteins, but interestingly, its absence impacts the transcription of several genes (16). There are important and challenging questions to address regarding whether ppGpp is a physiological ligand of HetC and whether its putative interaction with the whole HetC protein *in vivo* is important for the activity of the transporter. In *Anabaena cylindrica* ppGpp accumulates during the early stages of heterocyst differentiation (34). In *Nostoc*, the inactivation of the *relAana* gene which is required for ppGpp synthesis led to a mutant strain unable to form heterocysts and to grow under nitrogen-fixing conditions (35). The effect of ppGpp on heterocyst differentiation has been shown to be independent of the action of three factors known to be crucial for this process (*hetR*, *hetP,* and *hetZ* genes) (35). It is possible that ppGpp effect can be mediated by its action on HetC activity, and that following interaction with its ligand, HetC cleaves and exports regulatory peptides or proteins. The next crucial step in our understanding of the molecular mechanism underlining the regulatory role of HetC is the identification of its physiological substrate. The presence of a characteristic double-glycine leader peptide in the protein sequence of PCAT substrates allowed the identification of genes encoding putative PCAT substrates in several Gram-negative bacterial genomes. Since no gene encoding a protein with this characteristic leader peptide has been found near *hetC*, the genomic approach does not help identifying its potential substrates. Whether the gene encoding the substrate of HetC is located in the same cluster as *hetC* but encodes a protein with an atypical cleavage signal, or whether this gene is located elsewhere in the genome are open questions which are worth investigating. It has been suggested that HetC may be involved in the export of regulatory peptides such as PatS and HetN from differentiating cells (17). The proximity of *hetC* to *hetP*, a gene involved in the commitment step of heterocyst differentiation (36) suggests a potential functional relationship between their two products (HetC and HetP proteins). Furthermore, both HetC-GFP and HetP-GFP fusions were shown to locate near the heterocyst cell poles (17). The exact nature of the relationship between PatS, HetN, HetP, and HetC is still to be elucidated. It would be, for instance, interesting to uncover whether peptides derivatives from PatS/HetN or HetP can be exported by HetC.

If the challenge ahead is to reveal the nature of the substrate exported by HetC, a working model can be proposed based on our current knowledge of the differentiation process and the functioning of this class type of transporter. The cleaved substrate could generate an N-terminal extremity that would remain in the heterocyst and interact directly with a transcriptional regulator. The exported region could be perceived by a signal transduction system of neighboring vegetative cells, which would contribute to the inhibition of their candidacy for differentiation. This model would explain both the phenotype of the *hetC* deletion mutant where a stretch of several contiguous pro-heterocysts is observed (15) and the fact that HetC transcriptionally regulates several genes of the heterocyst (16). Unearthing the signaling pathway involving HetC will allow the identification for the first time in Gram-negative bacteria of a mechanism by which a PCAT regulates cell differentiation.

## MATERIALS AND METHODS

### Strains, plasmids, and primers

All the strains, plasmids, and primers used in this study are listed in file S1. All the cyanobacterial strains are derivatives of *Nostoc* PCC 7120 (Pasteur Cyanobacterial Collection, https://www.pasteur.fr/fr/sante-publique/crbip/les-collections/collection-cyanobacteries-pcc).

### Growth conditions, conjugation, and heterocyst induction

Unless otherwise indicated, *E. coli* and derivative strains were grown at 37°C in LB medium supplemented when appropriate with 50 µg mL^−1^ kanamycin, 100 µg mL^−1^ ampicillin, 50 µg mL^−1^ spectinomycin, and 30 µg mL^−1^ chloramphenicol. *Nostoc* derivative strains were grown at 28°C under constant illumination (40 µE m^−2^ s^−1^) in BG11 medium [blue-green algae medium (37)]; supplemented when appropriate with 2.5 µg mL^−1^ spectinomycin plus 2.5 µg mL^−1^ streptomycin. Heterocyst induction was performed by transferring mid-log phase cultures from BG11 to BG11_0_ (blue green algae medium without combined nitrogen source). Conjugation of *Nostoc* was performed using AM5501 *E. coli* strain transformed with the desired plasmid. Transformed AM5501 strains were grown until reaching optical density at 600 nm of 0.8 and 1 mL of cells suspension was washed three times with fresh LB medium. *Nostoc* receiver strains were grown until reaching optical density at 750 nm comprised between 0.8–1 and 4 mL were washed once with fresh BG11. Washed *Nostoc* and *E. coli* strains were then mixed, pelleted together, and resuspended in 300 µL of BG11 medium and then spotted on BG11 plates containing 5% (vol/vol) of LB. Plates were incubated for 24–48 h. The bacteria were then harvested and resuspended in 400 µL of BG11 medium. A volume of 350 and 50 µL of this suspension was spread on BG11 plates containing 2.5 µg mL^−1^ spectinomycin and 2.5 µg mL^−1^ streptomycin.

### Construction of the *hetC* deletion strain

The pCpf1-Δ*hetC* plasmid was introduced in *Nostoc* PCC 7120 wild-type strain by conjugation and exconjugants were tested by colony PCR using DelhetC-Fwd and Rev primers. Positive exconjugants were pricked out on BG11 plates supplemented with 2.5 µg mL^−1^ spectinomycin and 2.5 µg mL^−1^ streptomycin for eight successive generations and then cultured in liquid for genomic DNA (gDNA) extraction. The absence of the *hetC* gene and total segregation of the mutant was verified by PCR on gDNA and sequencing using DelhetC-Fwd and SeqDelhetC-Rev primers. Totally segregated clones were then plated on BG11 plates without antibiotics and then inoculated on BG11 plates supplemented with 10% saccharose to counter select pCpf1-Δ*hetC* plasmid cured clones. Loss of the editing plasmid was verified by colony PCR using pCpf1-Fwd and Rev primers and by plating the obtained strain on BG11 supplemented with 2.5 µg mL^−1^ spectinomycin and 2.5 µg mL^−1^ streptomycin. All the experiments were conducted on two independent clones.

### Microscopy

Observations were realized using NIKON ECLISE E800 optical microscope under ×100 magnifying objective with immersion oil. Images captures were realized with Still DXM 1200 NIKON digital camera controlled with ATC-1 software. Bright field observations were realized with white light, photosynthetic pigments-related fluorescence observations were realized using TRITC filter (Ex 540/25, Dm 565).

### Plasmid construction

All the primers used for plasmid constructs are listed in file S1, Table S3. All the cloning steps were carried out in DH5α *E. coli* strain, and In-fusion (Takara) steps were carried out using Cloning Enhancer-based method (Takara) according to the manufacturer instructions. All the PCR amplification steps were performed with CloneAmp (Takara) polymerase mix. All the recombinant plasmids were analyzed by sequencing.

#### Construction of pKTop derivative expression vectors

Full-length and shortened coding sequences of *hetC* were amplified from *Nostoc* genomic DNA (gDNA) using HetC-M1-Fwd common forward primer and HetC-L1044-Rev, HetC-Y486-Rev, HetC-N523-Rev, HetC-Q547-Rev, HetC-R604-Rev, HetC-S626-Rev, HetC-R717-Rev, or HetC-G738-Rev reverse primers. Amplified fragments were digested with XbaI and SacI restriction enzymes and then ligated into pKTop between XbaI and SacI restriction sites resulting, respectively, into pKTop-HetC, pKTop-HetC_1–486_, pKTop-HetC_1–523_, pKTop-HetC_1–547_, pKTop-HetC_1–604_, pKTop-HetC_1–626_, pKTop-HetC_1–717_, pKTop-HetC_1–738_ plasmids.

#### pCpf1-ΔhetC vector construction

pCpf1-Δ*hetC* vector was constructed as described in reference (38). A region of 1 kb upstream and downstream of the *hetC* gene were amplified from *Nostoc* gDNA using, respectively, RP-hetCup-Fwd/Rev and RP-hetCdown-Fwd/Rev primers and then inserted in pCpf1-Sp-ccdB between BamHI sites by In-fusion resulting in pCpf1-RP-hetC plasmid. The spacer sequence was designed using the ChopChop software (39) with the size of the primers set to 22 nucleotides and the 5′-PAM sequence designed as TTN (40). Spacer-hetC-Fwd and Rev primers were annealed at 95°C to obtain the *hetC* spacer, which was then ligated between the AarI sites of the pCpf1-RP-hetC plasmid resulting in pCpf1-ΔhetC plasmid.

#### hetC subcloning and mutagenesis

Coding sequence of *hetC* was amplified from *Nostoc* gDNA using, respectively, HetC-Fwd/Rev primers and then inserted between BamHI and EcoRI sites into pRL25SC by In-fusion resulting in pRL25SC-HetC plasmid.

Mutations in *hetC* were introduced by In-fusion site-directed mutagenesis on pRL25SC-HetC plasmid. *hetC* 347th cysteine, 420th histidine, and 970th glutamine coding codons were substituted by alanine codons using respectively HetC-C347A-Fwd/Rev, HetC-H420A-Fwd/Rev, and HetC-E970A-Fwd/Rev primer couples resulting in pRL25SC-HetC_C347A_, pRL25SC-HetC_H420A_, and pRL25SC-HetC_E970A_ plasmids, respectively. To obtain the pRL25SC-HetC_C347A/H420A_ plasmid, primers HetC-H420A-Fwd/Rev were used on the pRL25SC-HetC_C347A_ plasmid. Codons 2–278 of *hetC* were removed from pRL25SC-HetC using HetC-delcNMP-Fwd and Rev primers resulting in pRL25SC-HetC_ΔcNMP_ plasmid.

#### E. coli overproduction vector construction

DNA sequences encoding the first 480 residues of *hetC* were amplified from pRL25SC-HetC and pRL25SC-HetC_C347A/H420A_ plasmids using HetC-Nter-Fwd and Rev primers and then inserted into pET28-a between NcoI/XhoI restriction sites by In-fusion resulting, respectively, in pET28-HetC-wt and pET28-HetC-mut plasmids.

#### Nostoc-replicative vector construction

XbaI-NotI fragments containing the P*petE* promoter and *hetC* coding sequence were excised from pRL25SC-HetC, pRL25SC-HetC_C347A_, pRL25SC-HetC_H420A_, pRL25SC-HetC_C347A/H420A_, pRL25SC-HetC_E970A_, and pRL25SC-HetC_ΔcNMP_ plasmids then inserted into pRR between SpeI (compatible with XbaI) and NotI restriction sites resulting, respectively, in pRR-P*petE::hetC*, pRR-P*petE::hetC_C347A_*, pRR-P*petE::hetC_H420A_*, pRR-P*petE::hetC_C347A/H420A_*, pRR-P*petE::hetC_E970A_*, and pRR-P*petE::hetC*_ΔcNMP_ plasmids.

#### HetC topology determination

Competent *E. coli* DH5α cells were transformed with pKTop derivative plasmids and then spread on LB plates supplemented with 100 µg mL^−1^ X-Pho (5-bromo-4-chloro-3-indolyl phosphate disodium salt; Apollo Scientific), 100 µg mL^−1^ Red-Gal (5-chloro-3-inodyl-beta-d-galactopyranoside; Apollo Scientific) and 50 µg mL^−1^ kanamycin. The plates were incubated overnight at 30°C.

### Recombinant HetC_NTD_ purification

Hexa-his tagged HetC_NTD_-_wt_ and HetC_NTD_-_mut_ were produced in BL21(DE3) cells, respectively, transformed with pET28-HetC-wt and pET28-HetC-mut plasmids. Cells were grown at 37°C until reaching optical density at 600 nm of 0.5. Induction was performed with 0.2 mM IPTG for 3 h at 30°C. Bacteria were collected by centrifugation and broken using a French press (10,000 p.s.i.) in 10 mL of purification buffer (50 mM Tris-HCl, pH 8, 300 mM NaCl, 2% glycerol) supplemented with 0.5 mg mL^−1^ lysozyme (Merck) and 1 mg mL^−1^ of DNAseI (Merck). The lysate was cleared by ultracentrifugation and then supplemented with 10 mM of imidazole before loading on a 1 mL HiFliQ Ni-NTA FPLC column (Generon) using an ÄKTA Prime apparatus. Immobilized proteins were washed with 10 mL of purification buffer containing 10 mM imidazole then successive washing steps of 6 mL of purification buffer containing 20 mM, 30 mM, and 40 mM of imidazole. Elution was performed in 6 × 1 mL fractions of purification buffer containing 200 mM of imidazole, and elution fractions were analyzed by SDS-PAGE. Purified recombinant proteins were desalted using PD-10 buffer exchange columns (Cytiva) with cold buffer 50 mM HEPES, pH 7.3, 100 mM NaCl, 5% glycerol. Protein concentration was determined by spectrophotometry using Bio-Rad Protein Assay kit based on the method of Bradford according to manufacturer’s instructions.

### Sodium dodecyl sulfate–polyacrylamide gel electrophoresis (SDS-PAGE)

Proteins were separated on 4%–20% polyacrylamide gradient gels (NuSep). Whole cells were resuspended in LAEMMLI buffer (60 mM Tris-HCl, pH 6.8, 10% glycerol, 5% dithiothreitol, 2% sodium dodecyl sulfate, 0.005% bromophenol blue), heated at 100°C for 10 min, and loaded up to 0.2 absorbance units (600 nm) equivalent per well. A volume of 15 µL of purification fractions was mixed with 5 µL of 4× TS-TD buffer (200 mM Tris-HCl, pH 8.8, 1 M saccharose, 300 mM dithiothreitol, 18% sodium dodecyl sulfate, 5 mM EDTA, 0.025% bromophenol blue), heated at 100°C for 5 min, and loaded on SDS-PAGE gel. Electrophoresis was performed at 200 V for 50 min, and gels were stained with InstantBlue Coomassie protein stain (Expedeon).

### HetC_NTD_ peptidase activity determination

Standard reactions were realized in 96 wells plates (path length of 0.4 cm) in a final volume of 100 µL. Ten micrograms of purified HetC_NTD_-_wt_ or HetC_NTD_-_mut_ were preincubated for 30 min at 4°C with 20 mM of β-mercaptoethanol in 50 µL of reaction buffer (50 mM HEPES, pH 7.3, 100 mM NaCl, 5% glycerol). A volume of 25 µL of reaction buffer was added containing when indicated 4× of divalent cation (for a final concentration of 5 mM). The reaction was started by the addition of 25 µL of reaction buffer containing 4× of L-arginine *p*-nitroanilide (Sigma) protease substrate (1 mM final concentration) and incubated at 37°C for 5 h. The cleavage product (*p*-nitroaniline) was monitored at *t* = 0 h and *t* = 5 h at 405 nm using a Tecan Spark plate reader spectrophotometer, and background readings of hydrolysis of the substrate without HetC were subtracted. Specific activities were calculated with *ε*_405nm_ = 9.9 L mmol^−1^ cm^−1^.

### NanoDSF experiments

Purified N-terminal domain of HetC was analyzed by nano differential scanning fluorimetry (nanoDSF). Thermal denaturation assays were performed using the Prometheus NT.48 instrument and analyzed using PR.thermocontrol V2.0.4. software (Nanotemper technologies, DE). HetC_NTD_ was used at 10 µM (0.55 mg/mL) and supplemented with variable concentrations of compounds as specified in the figure legends. The capillaries were then filled with 10 µL of the sample mixture and placed on the sample holder. A temperature gradient of 1°C/min from 20 to 95°C was applied, and the intrinsic protein fluorescence at 330 and 350 nm was recorded. The ratio of fluorescence intensity at 350/330 nm was used to determine the melting temperatures.

### Isothermal titration calorimetry

Isothermal titration calorimetry (ITC) experiments were carried out at 25°C in a MicroCal iTC200 calorimeter (Malvern Panalytical) by injecting 2.5 mM ppGpp into 0.039 mM of wild-type HetC_NTD_. ITC buffer was composed of 50 mM HEPES pH 7.5, 100 mM NaCl, 5% glycerol. Initially, 0.4 µL was injected over 0.8 s followed by injections of 2 µL over 4 s, injections occurred every 150 s with a filter of 5 s, and the cell stirring speed was set at 750 rpm until the syringe was empty. For each titration, a control run with injectant and buffer alone in the cell was performed, and the resulting signal was subtracted from protein-nucleotide data. Binding affinity, stoichiometry, and thermodynamic parameters were obtained by nonlinear least-squares fitting of experimental data using a single-site binding model from the MicroCal PEAQ-ITC software (Malvern Panalytical).

### Statistical analysis

For the peptidase assays, the values presented correspond to the means of three independent assays containing each 10 independent colonies. The results are expressed as mean ± standard variation. A student *t*-test was used to compare the means of the different assays. *P value ≤ 0.05; **P value ≤ 0.01; ***P value ≤ 0.00.

### Bioinformatics analysis

Clustal Omega (41) was used to align the protein sequences. Functional domains were analyzed using pfam (42). The software used to predict the topology of HetC is listed in Fig. S1. Alphafold was used to predict the structure of HetC monomer (20).

## SUPPLEMENTAL MATERIAL

Supplemental material spectrum.04058-23-s0001.docx Tables S1 to S3; Fig. S1 to S3.

ASM does not own the copyrights to Supplemental Material that may be linked to, or accessed through, an article. The authors have granted ASM a non-exclusive, world-wide license to publish the Supplemental Material files. Please contact the corresponding author directly for reuse.
