# Supplementary material for: Unravelling HetC as a peptidase-based ABC exporter driving functional cell differentiation in the cyanobacterium Nostoc PCC 7120
Source: Microbiol Spectr. 2024 Feb 15;12(4):e04058-23. doi: 10.1128/spectrum.04058-23 (PMC10986499; doi:10.1128/spectrum.04058-23)
Supplement: Supplemental material — Tables S1 to S3; Fig. S1 to S3. [file spectrum.04058-23-s0001.docx]

**Supplementary file 1**

**Table S1: *E. coli* and *Nostoc* strains used in this study**

| **Strains** | **Description/genotype** | **Source/reference** |  |
| --- | --- | --- | --- |
| ***E. coli* strains** |  |  |  |
| DH5α | *fhuA2, lac(Δ)U169, phoA, glnV44, Φ80', lacZ(Δ)M15, gyrA96, recA1, relA1, endA1, thi-1, hsdR17* | (1) | |
| DH5α/pET28-a | DH5α strain containing the pET28-a plasmid | This study |  |
| DH5α/pKTop | DH5α strain producing the PhoA-LacZ fusion | This study |  |
| DH5α/pKTop-HetC | DH5α strain producing the HetC-PhoA-LacZ fusion | This study |  |
| DH5α/pKTop-HetC_1-486_ | DH5α strain producing the HetC_1-486_-PhoA-LacZ fusion | This study |  |
| DH5α/pKTop-HetC_1-523_ | DH5α strain producing the HetC_1-522_-PhoA-LacZ fusion | This study |  |
| DH5α/pKTop-HetC_1-547_ | DH5α strain producing the HetC_1-547_-PhoA-LacZ fusion | This study |  |
| DH5α/pKTop-HetC_1-604_ | DH5α strain producing the HetC_1-604_-PhoA-LacZ fusion | This study |  |
| DH5α/pKTop-HetC_1-626_ | DH5α strain producing the HetC_1-626_-PhoA-LacZ fusion | This study |  |
| DH5α/pKTop-HetC_1-717_ | DH5α strain producing the HetC_1-717_-PhoA-LacZ fusion | This study |  |
| DH5α/pKTop-HetC_1-738_ | DH5α strain producing the HetC_1-738_-PhoA-LacZ fusion | This study |  |
| BL21DE3 | *fhuA2 [lon], ompT, gal, (λ DE3), [dcm], ∆hsdS, λDE3 = λsBamHIo,* *∆EcoRI-int::(lacI::PlacUV5::T7 gene1), i21∆nin5* | [NEB](https://international.neb.com/products/c2527-bl21de3-competent-e-coli#Product%20Information) |  |
| BL21DE3/pET28 | BL21DE3 strain containing the unmodified pET28-a plasmid | This study |  |
| BL21DE3/pET28-HetC-wt | BL21DE3 strain containing the pET28-HetC-wt plasmid | This study |  |
| BL21DE3/pET28-HetC-mut | BL21DE3 strain containing the pET28-HetC-mut plasmid | This study |  |
| AM5501 | DH5α strain containing both pRL443 and pAM5505 plasmids for conjugation | (2) |  |
| ***Nostoc* strains** |  |  |  |
| *Nostoc* PCC 7120 | Wild type strain (WT) | [Pasteur collection](https://www.pasteur.fr/fr/sante-publique/crbip/les-collections/collection-cyanobacteries-pcc) |  |
| Δ*hetC* | Nostoc PCC 7120 cleanly deleted from *hetC*. | This study |  |
| Δ*hetC*/ pRR | Δ*hetC* strain containing the pRR001 plasmid | This study |  |
| Δ*hetC*/ HetC | Δ*hetC* strain containing the pRR-P*petE*::*hetC* plasmid | This study |  |
| Δ*hetC*/ HetC_C347A/H420A_ | Δ*hetC* strain containing the pRR-P*petE*::*hetC_C347A_*_/_*_H420A_* plasmid | This study |  |
| Δ*hetC*/ HetC_E970A_ | Δ*hetC* strain containing the pRR-P*petE*::*hetC_E970A_* plasmid | This study |  |
| Δ*hetC*/ HetC_ΔcNMP_ | Δ*hetC* strain containing the pRR-P*petE*::*hetC*_ΔcNMP_ plasmid | This study |  |

**Table S2: Plasmids used in this study**

| **Plasmids** | **Description** | **Source/reference** |
| --- | --- | --- |
| pKTop | Kan^R^. Contains a dual reporter *pho*-*lac* for protein topology determination. | (3) |
| pKTop-HetC | Kan^R^. pKTop derivative, the *pho*-*lac* dual reporter is fused in 5’ with full-length *hetC* coding sequence. | This study |
| pKTop-HetC_1-486_ | Kan^R^. pKTop derivative, the *pho*-*lac* dual reporter is fused in 5’ with partial *hetC* coding sequence encoding its first 486 residues. | This study |
| pKTop-HetC_1-523_ | Kan^R^. pKTop derivative, the *pho*-*lac* dual reporter is fused in 5’ with partial *hetC* coding sequence encoding its first 523 residues. | This study |
| pKTop-HetC_1-547_ | Kan^R^. pKTop derivative, the *pho*-*lac* dual reporter is fused in 5’ with partial *hetC* coding sequence encoding its first 547 residues. | This study |
| pKTop-HetC_1-604_ | Kan^R^. pKTop derivative, the *pho*-*lac* dual reporter is fused in 5’ with partial *hetC* coding sequence encoding its first 604 residues. | This study |
| pKTop-HetC_1-626_ | Kan^R^. pKTop derivative, the *pho*-*lac* dual reporter is fused in 5’ with partial *hetC* coding sequence encoding its first 626 residues. | This study |
| pKTop-HetC_1-717_ | Kan^R^. pKTop derivative, the *pho*-*lac* dual reporter is fused in 5’ with partial *hetC* coding sequence encoding its first 717 residues. | This study |
| pKTop-HetC_1-738_ | Kan^R^. pKTop derivative, the *pho*-*lac* dual reporter is fused in 5’ with partial *hetC* coding sequence encoding its first 738 residues. | This study |
| pRL25SC | pRL25T derivative containing the copper inducible P*petE* promoter | Lab collection |
| pRL25SC-HetC | Kan^R^. pRL25SC derivative with *hetC* native coding sequence under P*petE* promoter control | This study |
| pRL25SC-HetC_C347A_ | Kan^R^. pRL25SC derivative with *hetC_C347A_* coding sequence under P*petE* promoter control | This study |
| pRL25SC-HetC_H420A_ | Kan^R^. pRL25SC derivative with *hetC_H420A_* coding sequence under P*petE* promoter control | This study |
| pRL25SC-HetC_C347A/H420A_ | Kan^R^. pRL25SC derivative with *hetC_C347A/H420A_* coding sequence under P*petE* promoter control | This study |
| pRL25SC-HetC_E970A_ | Kan^R^. pRL25SC derivative with *hetC_E970A_* coding sequence under P*petE* promoter control | This study |
| pRL25SC-HetC_ΔcNMP_ | Kan^R^. pRL25SC derivative with *hetC*_ΔcNMP_ coding sequence under P*petE* promoter control | This study |
| pET28-a | Kan^R^. For his-tagged protein production in *E.coli* | Novagen® |
| pET28-HetC-wt | Kan^R^. pET28-a derivative, for the production of C-terminally 6his-tagged HetC_1-480_ (HetC_NTD-wt_) | This study |
| pET28-HetC-mut | Kan^R^. pET28-a derivative, for the production of C-terminally 6his-tagged HetC_1-480_ with C_347_→A and H_420_→A substitutions (HetC_NTD-mut_) | This study |
| pAM5505 | Cm^R^. pRL623 derivative helper plasmid for conjugation | Addgene (#[132664](https://www.addgene.org/132664/))  (2) |
| pRL443 | Amp^R^. RT4 derivative plasmid for conjugation | Addgene (#[70261](https://www.addgene.org/70261/))  (2) |
| pCpf1-Sp-ccdB | Sp/Sm^R^. Replicative in *Nostoc*, Cpf1-based CRISPR plasmid with CcdB and SacB counter selection markers. | (4) |
| pCpf1-RP-*hetC* | Sp/Sm^R^. pCpf1-Sp-ccdB derivative with *hetC* repair template | This study |
| pCpf1-Δ*hetC* | Sp/Sm^R^. pCpf1-RP-hetC derivative for *hetC* clean deletion | This study |
| pRR001 | Sp/Sm^R^. Replicative in *Nostoc* | (5) |
| pRR-P*petE*::*hetC* | Sp/Sm^R^. pRR001 derivative with P*petE*::*hetC* construct | This study |
| pRR-P*petE*::*hetC_C347A_* | Sp/Sm^R^. pRR001 derivative with P*petE*::*hetC_C347A_* construct | This study |
| pRR-P*petE*::*hetC_H420A_* | Sp/Sm^R^. pRR001 derivative with P*petE*::*hetC_H420A_* construct | This study |
| pRR-P*petE*::*hetC_C347A/H420A_* | Sp/Sm^R^. pRR001 derivative with P*petE*::*hetC_C347A/H420A_* construct | This study |
| pRR-P*petE*::*hetC_E970A_* | Sp/Sm^R^. pRR001 derivative with P*petE*::*hetC_E970A_* construct | This study |
| pRR-P*petE*::*hetC*_ΔcNMP_ | Sp/Sm^R^. pRR001 derivative with P*petE*::*hetC*_ΔcNMP_ construct | This study |

**Table S3: Primers used in this study**

| **Primers** | **5’-3’ Sequence** |
| --- | --- |
| **pKTop derivative expression vectors construction** | |
| HetC-M1-Fwd | AATTTCTAGATAATCCCTCTTCGTCGTTAAGAG |
| HetC-L1044-Rev | AATTGAGCTCGATAGTTGCAGTTGAGCTAAGTGG |
| HetC-Y486-Rev | AATTGAGCTCGAATAATTCCACAATGTTTGCCC |
| HetC-N523-Rev | AATTGAGCTCGAGTTTTTGATAGGCATTACTTGGTCG |
| HetC-Q547-Rev | AATTGAGCTCGACTGCGCCGTTAACACAGTC |
| HetC-R604-Rev | AATTGAGCTCGACCTACGGCTAATAAACAGTTG |
| HetC-S626-Rev | AATTGAGCTCGAACTGTAATAGGTCATCAACCC |
| HetC-R717-Rev | AATTGAGCTCGACCGCGTCACTTGTAAACGATG |
| HetC-G738-Rev | AATTGAGCTCGAACCCATCACCAAAGTAGCC |
| ***hetC* cloning** | |
| HetC-Fwd | CGATGGATCCTTAGGAGAAATACCATGAATCCCTCTTCGTCG |
| HetC-Rev | AGTAGAATTCCTATAGTTGCAGTTGAGCTAAG |
| HetC-Nter-Fwd | AGGAGATATACCATGAATCCCTCTTCGTCGTTAAGAGT |
| HetC-Nter-Rev | GGTGGTGGTGCTCGAGACCTTGAAAATAAAGATTTTCCCCATAGCGACCAAGAGA |
| ***hetC* mutagenesis** | |
| HetC-C347A-Fwd | ATCAGACGCGGGTGCAGCTTGTTTGGCG |
| HetC-C347A-Rev | GCACCCGCGTCTGATGAACTCTGTTGTTGAA |
| HetC-H420A-Fwd | AGGAAATGCGTATGTAGTAGTGTGGCAGATTAAAG |
| HetC-H420A-Rev | ACATACGCATTTCCTTGCCAGTGAGCA |
| HetC-E970A-Fwd | TTTAGATGCGGCCACTAGCGGTTTAGAT |
| HetC-E970A-Rev | GTGGCCGCATCTAAAATCAAAATTTTGGGAT |
| HetC-delcNMP-Fwd | ATACCATGGCGCCCGTTGAGGTGGTTA |
| HetC-delcNMP-Rev | CGGGCGCCATGGTATTTCTCCTAAGGATCCAT |
| **CRISPR/Cpf1 plasmid construction** | |
| Spacer-hetC-Fwd | AGATGCCTCCCGCCAGGTAGATGATA |
| Spacer-hetC-Rev | AGACTATCATCTACCTGGCGGGAGGC |
| RP-hetCup-Fwd | ATATCTAGATCTCATGGATCCATGGGAATGAAAGACGAAGGTTT |
| RP-hetCup-Rev | ACTGACAACTAGTTTAATTTCTGTTTGGTGTGT |
| RP-hetCdown-Fwd | AAATTAAACTAGTTGTCAGTCGTCAATAGTTTT |
| RP-hetCdown-Rev | CGTTGTTGCCATTGCGGATCCGGTTGTTATTGTTTTGGATGAAAA |
| ***hetC* deletion strain verification** | |
| DelhetC-Fwd | TTCAGTAAAGTTTGAGGAAATACCG |
| DelhetC-Rev | TGTCAGTTTTGCGATTTTATGC |
| SeqDelhetC-Rev | TTGAGGTAGGGACTGGCAAC |
| pCpf1-Fwd | ATTTAGGCAAAAACGGG |
| pCpf1-Rev | GGCACAGGAATAGTAGCATC |


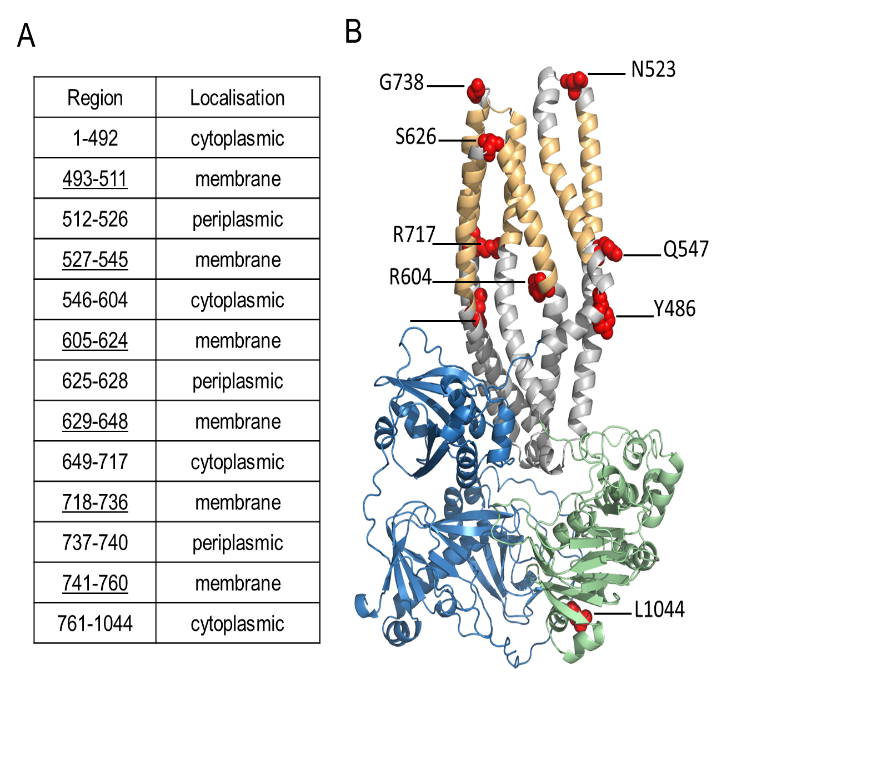


**Figure S1**. **Prediction of HetC topology.** **A**. Topology prediction using CCTOP. The boundaries of predicted HetC regions are listed in the table. **B**. Mapping of the HetC predicted TMHs onto the 3D-model generated by Alphafold. The N-domain comprising the cNMP and the peptidase subdomains is colored in blue, TMHs are in orange, the cytoplasmic and periplasmic extension of TMHs in gray and the ATPase domain in green. The residues where the fusion proteins were introduced (see legend of Figure 1) are shown in red spheres.


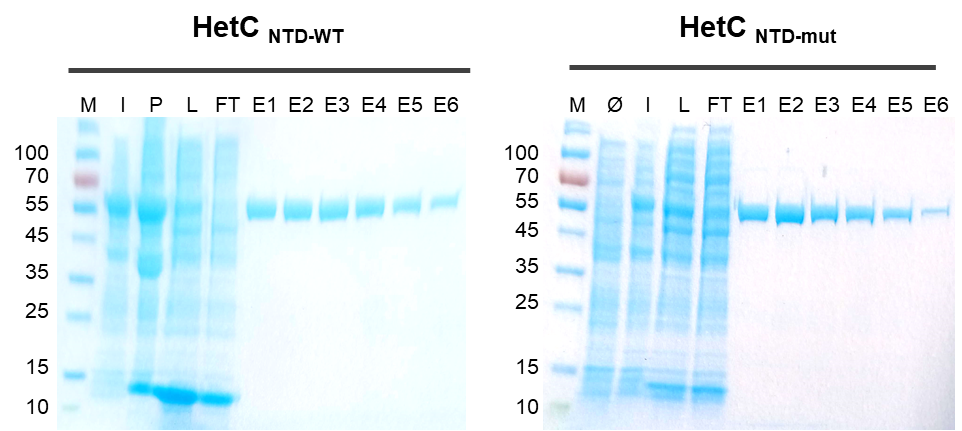


**Figure S2**. **SDS-PAGE of recombinant N-terminal region of HetC _NTD-wt_ protein (left) and HetC _NTD-mut_ protein (right) purification.** M, size marker (in kDa); Ø and I, whole BL21DE3 cells lysates respectively containing pET28-a unmodified or coding for 6his-HetCN-ter plasmids (wt, left; C_347_A / H_420_A double mutant, right); P, pellet; L, loading material; FT, flow-through; E_x_, elution fractions from the nickel-affinity chromatography.

**
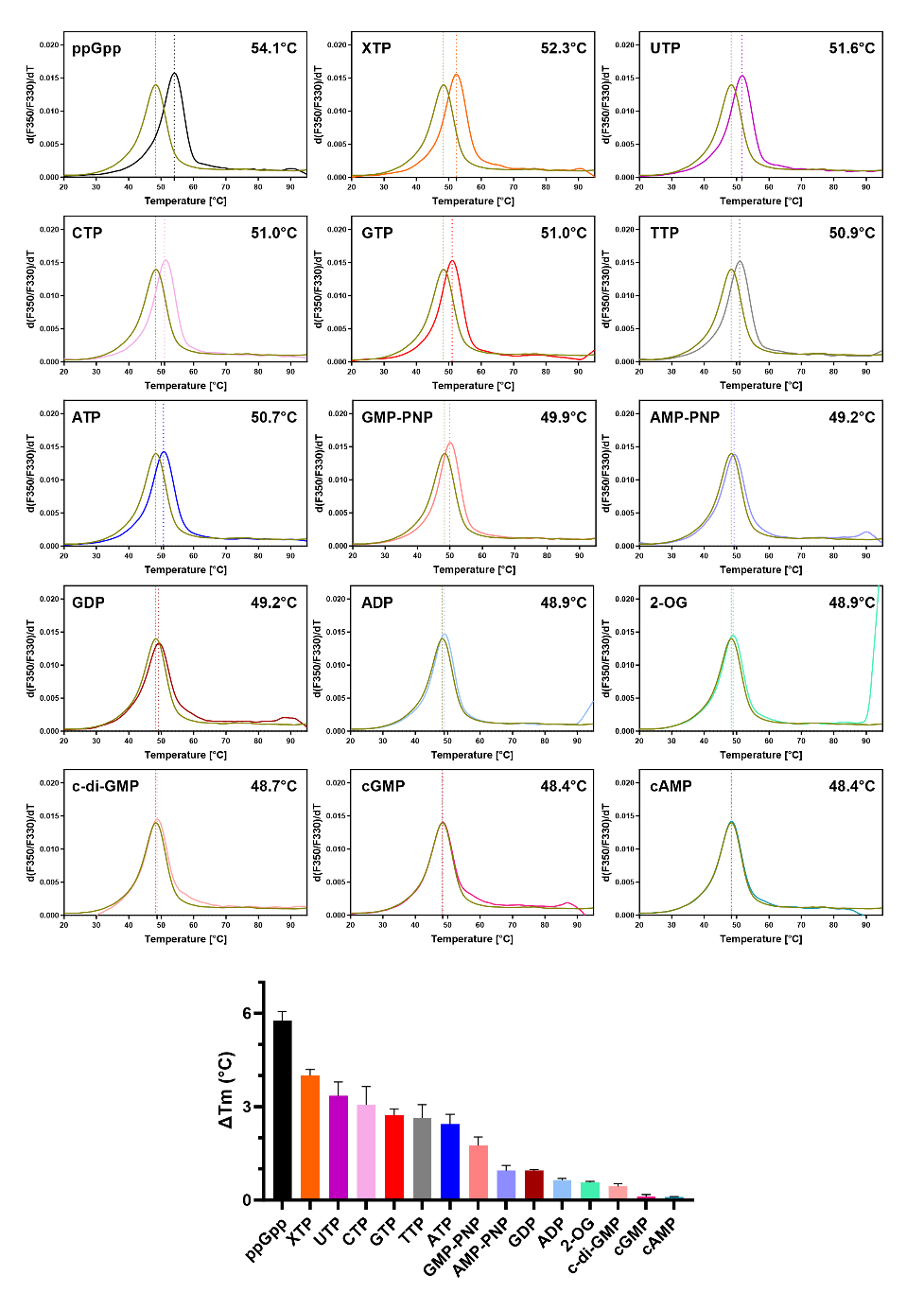
**

**Figure S3. Thermostability analysis of wild-type HetC_NTD_ in the presence of different effectors (2 mM) probed by differential scanning fluorimetry**. The first-derivatives curves of the ratio of fluorescence emitted at 350 and 330 nm with Tm in °C (top panels) and the ∆Tm in °C compared to the HetC_NTD_ Tm of 48.3°C (bottom panels) are shown. In each graph, the curve of HetC_NTD_ in the apo state is colored in olive green

**References**

1. Taylor RG, Walker DC, McInnes RR. 1993. E. coli host strains significantly affect the quality of small scale plasmid DNA preparations used for sequencing. Nucleic Acids Res 21:1677-8.

2. Bishe B, Taton A, Golden JW. 2019. Modification of RSF1010-Based Broad-Host-Range Plasmids for Improved Conjugation and Cyanobacterial Bioprospecting. iScience 20:216-228.

3. Karimova G, Robichon C, Ladant D. 2009. Characterization of YmgF, a 72-residue inner membrane protein that associates with the Escherichia coli cell division machinery. J Bacteriol 191:333-46.

4. Menestreau M, Rachedi R, Risoul V, Foglino M, Latifi A. 2022. The CcdB toxin is an efficient selective marker for CRISPR-plasmids developed for genome editing in cyanobacteria. MicroPubl Biol 2022.

5. Rachedi R, Risoul V, Scholivet A, Foglino M, Latifi A. 2023. Evidence that the PatB (CnfR) factor acts as a direct transcriptional regulator to control heterocyst development and function in the cyanobacterium Nostoc PCC 7120. Mol Microbiol 119:492-504.
